# Total parathyroidectomy versus total parathyroidectomy with autotransplantation for refractory secondary hyperparathyroidism: short-term biochemical outcomes and postoperative calcium supplementation burden

**DOI:** 10.1186/s12902-026-02430-8

**Published:** 2026-07-21

**Authors:** Horng-Shiuan See, Kian-Hwee Chong, Yu-Tien Chen, Jia-Hui Chen, Chieh-Wen Lai

**Affiliations:** 1 Department of Surgery, Taipei Tzu Chi Hospital, Buddhist Tzu Chi Medical Foundation, New Taipei City, Taiwan; 2 School of Medicine, Tzu Chi University, Hualien, Taiwan; 3 Division of General Surgery, Department of Surgery, Taipei Tzu Chi Hospital, Buddhist Tzu Chi Medical Foundation, No.289, Jianguo Road, Xindian City, New Taipei City, Taiwan (R.O.C.)

**Keywords:** Secondary hyperparathyroidism, Total parathyroidectomy, Autotransplantation, Calcium supplementation, Chronic kidney disease

## Abstract

**Background:**

Comparative studies of total parathyroidectomy (TPTX) and total parathyroidectomy with autotransplantation (TPTX + AT) have primarily emphasized biochemical control and recurrence, whereas longitudinal outpatient oral calcium requirements have been less frequently quantified. This study compared short-term biochemical outcomes and postoperative calcium supplementation burden after these two procedures for refractory secondary hyperparathyroidism (SHPT).

**Methods:**

We retrospectively analyzed a nonrandomized cohort of 80 patients with refractory SHPT who underwent parathyroidectomy between January and December 2021 (TPTX, *n* = 40; TPTX + AT, *n* = 40). Biochemical markers were evaluated preoperatively and at postoperative day 3 and at postoperative months 1, 3, and 6. The practical postoperative outcome was prescribed oral calcium carbonate burden (500-mg tablets/day) at discharge and at 3 and 6 months. Acute intravenous calcium use and calcitriol supplementation were also recorded.

**Results:**

Both groups achieved satisfactory short-term biochemical control without major perioperative complications. Operative time was longer in the TPTX + AT group (*p* = 0.015). Compared with the TPTX + AT group, the TPTX group had higher serum calcium at 3 and 6 months, lower phosphate at 1 and 3 months, and lower bone-specific alkaline phosphatase and intact parathyroid hormone at 3 and 6 months. Despite these biochemical differences, acute intravenous calcium use and outpatient oral calcium and calcitriol supplementation did not differ significantly between groups.

**Conclusions:**

TPTX and TPTX + AT yielded satisfactory short-term biochemical outcomes. TPTX + AT was associated with higher postoperative intact parathyroid hormone levels, compatible with greater postoperative parathyroid activity; however, no significant between-group difference in prescribed oral calcium or calcitriol supplementation burden through 6 months was detected. Because procedure allocation was nonrandomized, thymectomy practices differed, and follow-up was limited to 6 months, these findings are descriptive and do not establish procedural superiority or long-term recurrence risk.

**Supplementary Information:**

The online version contains supplementary material available at https://doi.org/10.1186/s12902-026-02430-8.

## Introduction

The management of secondary hyperparathyroidism (SHPT) in patients with chronic kidney disease (CKD) is a considerable clinical challenge because of its multifactorial pathophysiology and substantial impact on patient outcomes [1]. SHPT is characterized by excessive parathyroid hormone (PTH) secretion and disturbances in calcium, phosphate, and vitamin D homeostasis [2]. These abnormalities contribute to renal osteodystrophy, vascular calcification, and increased morbidity and mortality in patients receiving dialysis [3].

Calcimimetics and vitamin D receptor activators are central components of medical therapy; however, treatment may be insufficient or poorly tolerated in a subset of patients [4]. Surgical intervention is considered for medically refractory SHPT, particularly when severe biochemical abnormalities or complications such as pruritus, calciphylaxis, progressive bone pain, osteitis fibrosa cystica, or pathologic fracture persist [5–7].

Subtotal parathyroidectomy (SPTX), TPTX, and TPTX + AT are established surgical options. Earlier studies reported substantial recurrence after SPTX [8], but contemporary evidence demonstrates that recurrence and complication rates depend on patient selection, remnant size, operative technique, and follow-up duration. A meta-analysis and a recent institutional cohort reported acceptable outcomes after carefully performed SPTX, indicating that SPTX should not be characterized uniformly as a high-recurrence procedure [9, 10].

TPTX seeks maximal removal of hyperfunctioning tissue, whereas TPTX + AT preserves an accessible source of parathyroid tissue. Cervical thymectomy may be performed routinely in some centers or selectively when ectopic or supernumerary glands are suspected; therefore, thymectomy practice is not uniform [11–14]. Both excessive suppression of PTH and persistent parathyroid activity have potential consequences. Marked PTH suppression may be associated with low-turnover bone disease, including adynamic bone disease, whereas residual or grafted tissue may contribute to persistent or recurrent hyperparathyroidism [1, 12–14].

Postoperative hypocalcemia frequently requires intravenous calcium during hospitalization and oral calcium plus calcitriol after discharge. Most previous reports have focused on biochemical outcomes, hungry bone syndrome, recurrence, or total inpatient calcium use [14, 15]. The actual outpatient oral calcium dose at serial follow-up visits represents a practical medication burden for patients but has been less consistently reported.

The present study therefore compared the short-term biochemical outcomes of TPTX and TPTX + AT in a real-world, nonrandomized cohort and specifically quantified prescribed oral calcium carbonate burden at discharge and at 3 and 6 months. The study was designed to describe postoperative management needs rather than to establish the superiority of either operation or to evaluate long-term recurrence.

## Materials and methods

### Study design and population

This retrospective study was approved by the Institutional Review Board of Buddhist Tzu Chi General Hospital, Taipei branch (No. 14-IRB068). Eighty patients with CKD-associated SHPT who underwent parathyroidectomy between January and December 2021 were included. Surgical indications included persistent iPTH elevation (> 800 pg/mL) with hypercalcemia or hyperphosphatemia despite medical treatment, intractable pruritus, calciphylaxis, progressive bone pain, osteitis fibrosa cystica, or pathologic fracture.

Preoperative localization included neck ultrasonography and Tc-99 m sestamibi scintigraphy. Computed tomography or magnetic resonance imaging was obtained when an ectopic gland was suspected. No ectopic parathyroid gland was identified on preoperative ultrasonography or sestamibi scintigraphy in this cohort. All procedures were performed by the same surgeon (C.-W.L.).

### Surgical strategy and procedure allocation

Patients underwent TPTX (*n* = 40) or TPTX + AT (*n* = 40). In the TPTX group, cervical thymectomy was not performed routinely and was reserved for suspected ectopic or supernumerary tissue. Patients in this group generally were not pursuing kidney transplantation because of comorbidity burden, age-related candidacy considerations, socioeconomic factors, or personal preference. In the TPTX + AT group, routine cervical thymectomy was performed, and approximately 40–50 mg of relatively healthy-appearing parathyroid tissue was implanted in the subcutaneous tissue of the anterior chest wall. Four parathyroid glands were identified and resected in all patients, with histopathologic confirmation. This confirmation did not exclude the possible presence of an unrecognized supernumerary, ectopic, or microscopic parathyroid focus. Procedure selection was not randomized and reflected shared decision-making incorporating anticipated transplant candidacy, comorbidity profile, and patient preference. Accordingly, the comparison represents two real-world surgical strategies that differed in both autotransplantation and thymectomy practice.

### Data collection and postoperative supplementation

Baseline variables included age, sex, dialysis modality, operative time, and hospital length of stay. Serum total calcium, phosphate, bone-specific alkaline phosphatase (BALP), and iPTH were measured preoperatively and at postoperative day 3 and at postoperative months 1, 3, and 6. During hospitalization, hypocalcemia was treated with intravenous calcium chloride (5.44 mEq/ampule) as clinically indicated on the basis of symptoms and serial serum calcium measurements. After discharge, oral calcium carbonate was prescribed using a 500-mg tablet formulation, and all patients received calcitriol (0.25 micrograms per dose). Oral calcium and calcitriol were titrated according to serial calcium measurements and clinical assessment. Postoperative oral calcium supplementation burden was defined as the prescribed number of calcium carbonate tablets per day at discharge and at 3 and 6 months. The available retrospective medication records supported analysis of the labeled tablet formulation and tablet count but not a uniform conversion to elemental calcium. Calcitriol dose and acute intravenous calcium use were also recorded.

### Statistical analysis

Continuous variables are presented as means ± standard deviations and categorical variables as frequencies (%). Between-group comparisons at each time point were performed using the Mann-Whitney U test for continuous variables and the chi-square test or Fisher’s exact test for categorical variables. Analyses were performed using SPSS version 18.0 (IBM SPSS Inc., Chicago, IL, USA), with statistical significance set at *p* < 0.05. Because these were exploratory time-point comparisons without adjustment for multiple testing, individual p values should be interpreted descriptively rather than as evidence of a causal treatment effect.

## Results

### Patient characteristics

A total of 80 patients (46 women and 34 men) were included and evenly divided between the TPTX and TPTX + AT groups. Baseline characteristics are summarized in Table 1. The mean age was 54 years. No statistically significant differences were detected in the measured baseline characteristics. Most patients received hemodialysis (86% overall); the proportion was 95.0% in the TPTX group and 77.5% in the TPTX + AT group (*p* = 0.094). No patient underwent kidney transplantation during the 6-month follow-up period.

**Table 1:** Baseline clinicopathologic characteristics of patients undergoing total parathyroidectomy only (No-AT) and total parathyroidectomy with autotransplantation (AT)

	Total (*N* = 80)	Group		*p*
		No-AT (*n* = 40)	AT (*n* = 40)	
Gender				0.715
female	46 (57.5%)	24 (60.0%)	22 (55.0%)	
male	34 (42.5%)	16 (40.0%)	18 (45.0%)	
PD/HD				
PD	11 (13.8%)	2 (5.0%)	9 (22.5%)	0.094
HD	69 (86.2%)	38 (95.0%)	31 (77.5%)	
Age (years)	57.40 ± 11.35	59.12 ± 10.15	54.58 ± 12.28	0.132
OP time (min)	76.32 ± 33.97	65.72 ± 30.20	86.92 ± 34.78	0.015 ^*^
Implant (g)	42.80 ± 11.19			
Hospital stay (day)	3.06 ± 0.62	2.96 ± 0.45	3.16 ± 0.75	0.355

Data are presented as means ± standard deviations or numbers (percentages)

*p < 0.05 between groups

No-AT = total parathyroidectomy only; AT = total parathyroidectomy with autotransplantation; PD = peritoneal dialysis; HD = hemodialysis; OP = operation

In the TPTX + AT group, the mean implanted parathyroid tissue weight was 42.8 mg. Operative time was longer in the TPTX + AT group than in the TPTX group (86.92 ± 34.78 vs. 65.72 ± 30.20 min, *p* = 0.015). No recurrent laryngeal nerve injury, hemorrhage, or hematoma occurred. Preoperative localization identified no ectopic gland (0/80). Four parathyroid glands were identified and resected in every patient and confirmed histopathologically. The indications for surgery are summarized in Table S1.

### Serum calcium levels

**Fig. 1:**
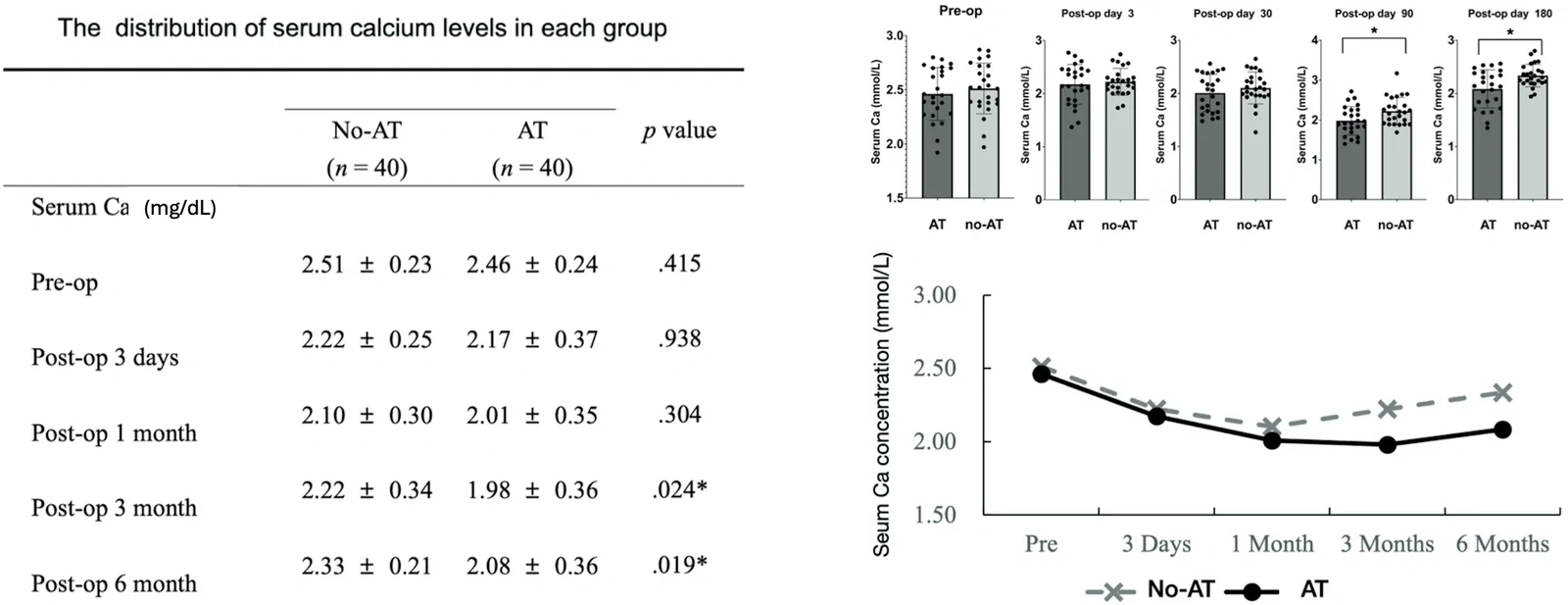
Serum calcium concentrations in the AT and No-AT groups at each time point. (**A**) Individual and mean serum calcium levels (mg/dL). *p < 0.05 between groups. (**B**) Mean serum calcium trends over time. AT = autotransplantation; No-AT = total parathyroidectomy only

Preoperative serum calcium levels were comparable between groups (Fig. 1). Postoperatively, serum calcium was higher in the TPTX group at 3 months (9.0 ± 1.3 vs. 8.0 ± 1.4 mg/dL, *p* = 0.024) and 6 months (9.4 ± 0.8 vs. 8.4 ± 1.4 mg/dL, *p* = 0.019). Because calcium and calcitriol doses were actively titrated during follow-up, these values represent treatment-adjusted serum calcium and should not be interpreted as a direct measure of endogenous calcium homeostasis.

### Serum phosphate levels

**Fig. 2:**
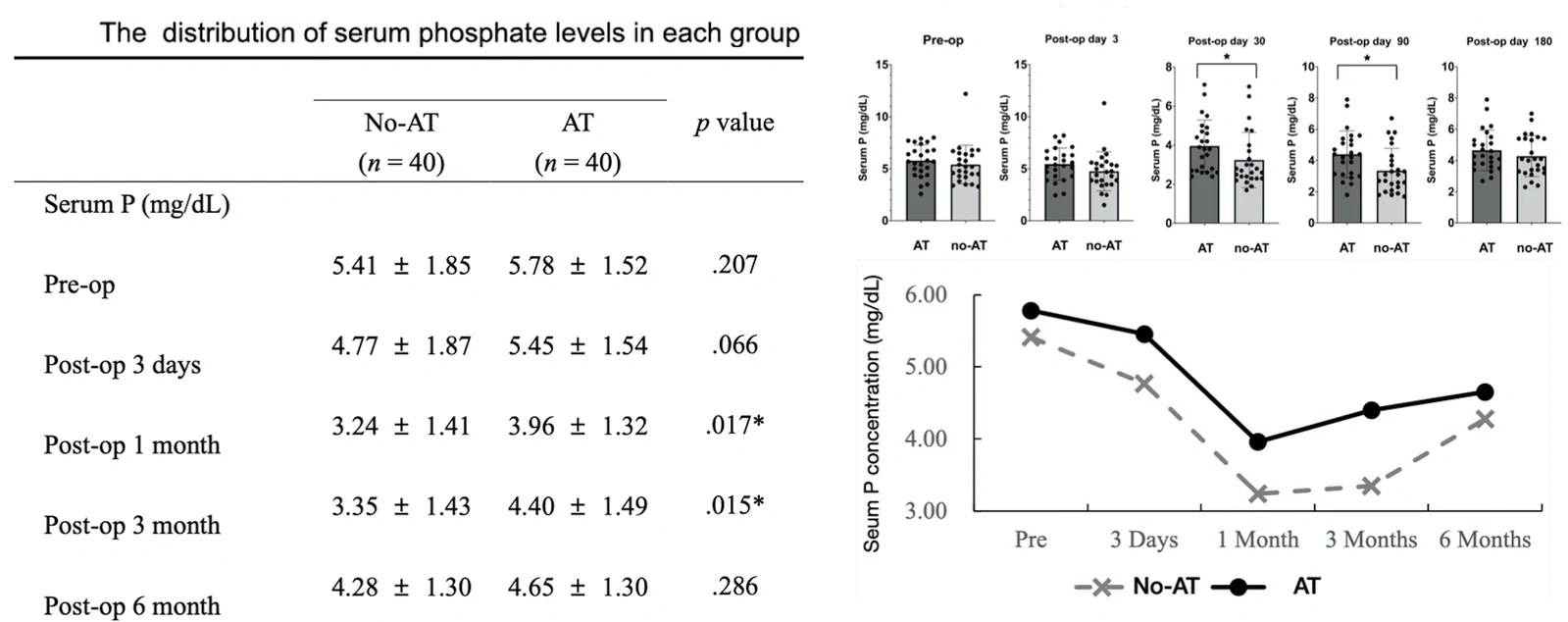
Serum phosphate concentrations in the AT and No-AT groups before and after surgery.(**A**) Bar graphs presenting serum phosphate (mg/dL) at each time point. Each dot represents one patient; bars indicate means ± SDs. *p < 0.05 between groups.(**B**) Line graph illustrating changes in mean serum phosphate levels over time for both groups.AT = autotransplantation; No-AT = total parathyroidectomy only

Preoperative serum phosphate levels were comparable between groups (Fig. 2). Postoperative phosphate was lower in the TPTX group at 1 month (3.24 ± 1.41 vs. 3.96 ± 1.32 mg/dL, *p* = 0.017) and 3 months (3.35 ± 1.43 vs. 4.40 ± 1.49 mg/dL, *p* = 0.015).

### Serum BALP levels

**Fig. 3:**
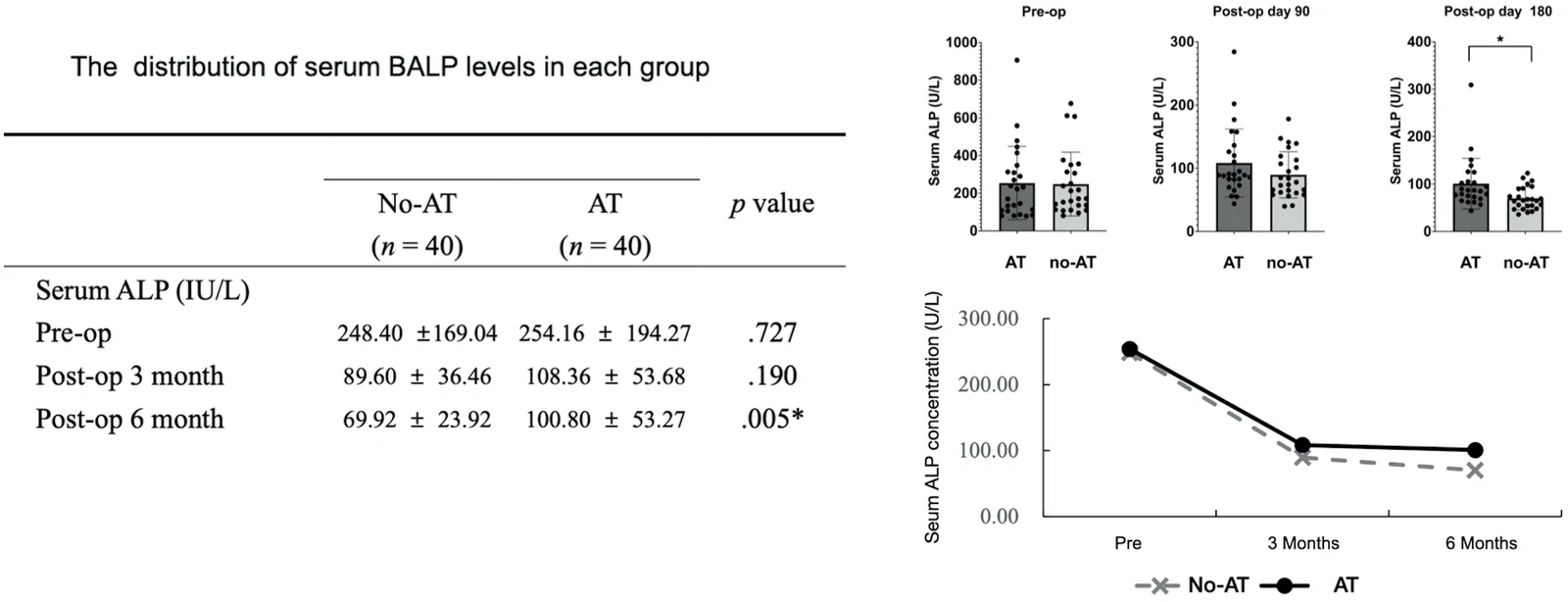
Serum bone-specific alkaline phosphatase (BALP) levels in the AT and No-AT groups before and after surgery. (**A**) Individual and mean BALP values (IU/L) at each time point. *p < 0.05 between groups. (**B**) Mean BALP trends over time. AT = autotransplantation; No-AT = total parathyroidectomy only

Preoperative BALP levels were similar between groups (Fig. 3). BALP was lower in the TPTX group at 3 months (89.60 ± 36.46 vs. 108.36 ± 53.68 IU/L, *p* = 0.019) and 6 months (69.92 ± 23.92 vs. 100.80 ± 53.27 IU/L, *p* = 0.005). These findings indicate different short-term bone-turnover marker profiles but do not establish a diagnosis of adynamic bone disease.

### Serum iPTH levels

**Fig. 4:**
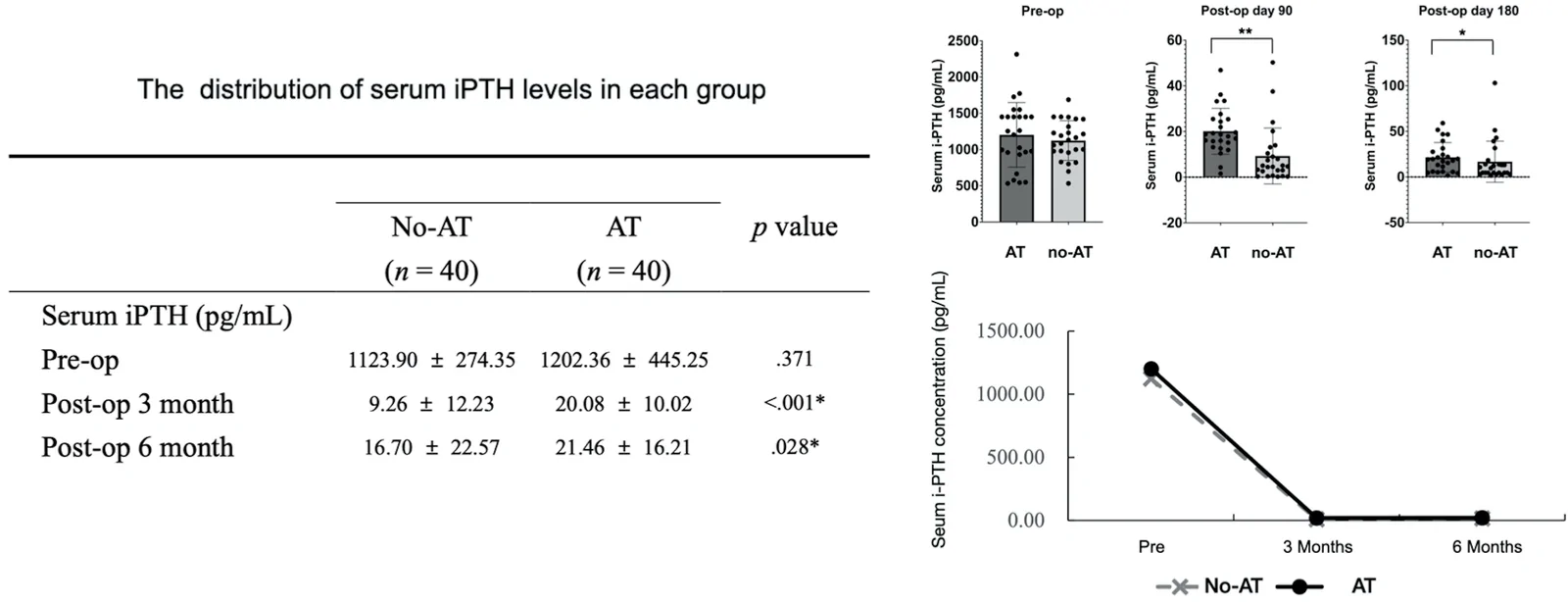
Serum iPTH (intact parathyroid hormone) levels in the AT and No-AT groups at baseline and after surgery. (**A**) Bar graphs presenting individual and mean iPTH values at each time point. *p < 0.05, **p < 0.01. (**B**) Line graph illustrating changes in mean iPTH levels over time. AT = autotransplantation; No-AT = total parathyroidectomy only

Preoperative iPTH levels were comparable between groups (Fig. 4). Postoperative iPTH was lower in the TPTX group at 3 months (9.26 ± 12.23 vs. 20.08 ± 10.02 pg/mL, *p* < 0.001) and 6 months (16.70 ± 22.57 vs. 21.46 ± 16.21 pg/mL, *p* = 0.028). At 6 months, the mean iPTH level in each group was within or near the laboratory reference range of 15–65 pg/mL; individual values varied.

### Postoperative calcium supplementation burden

**Fig. 5:**
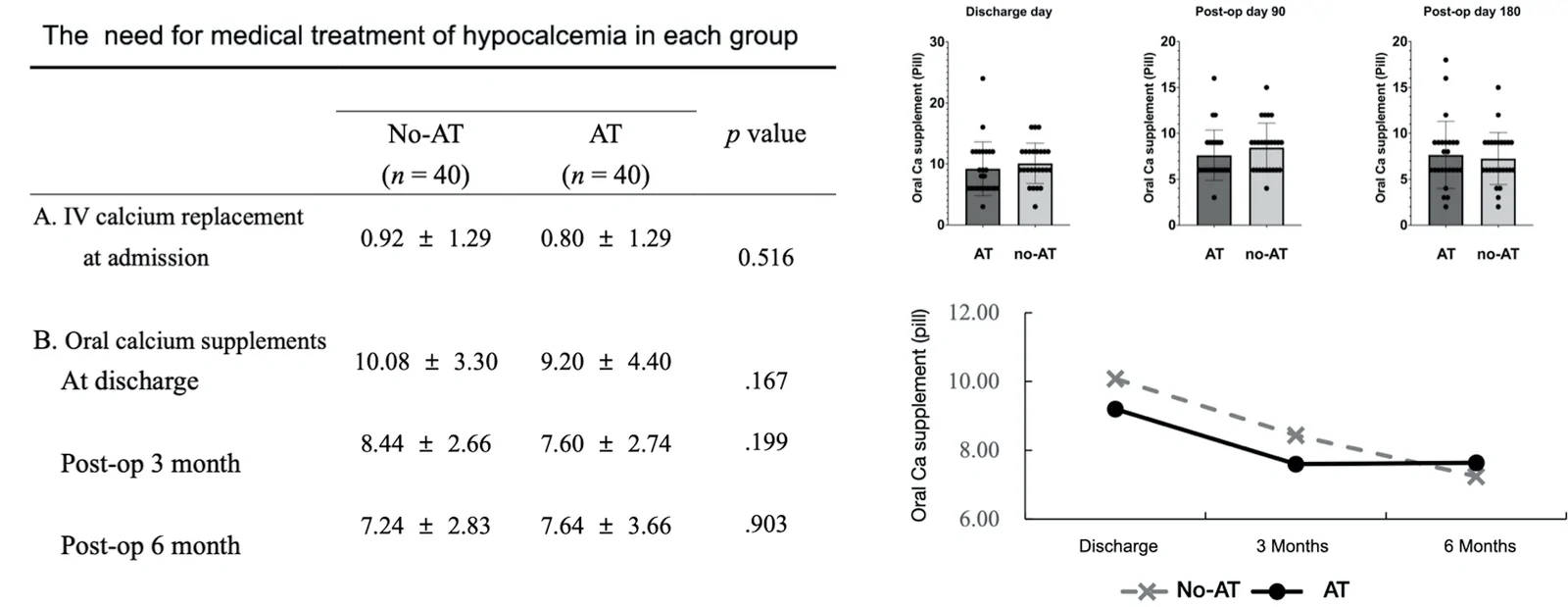
Prescribed oral calcium carbonate supplementation burden (500-mg tablets/day, as recorded in medication orders) in the TPTX (No-AT) and TPTX + AT (AT) groups at discharge and at 3 and 6 months after surgery. All patients received calcitriol; no significant between-group difference in calcitriol dose was detected. The displayed tablet count reflects prescribed postoperative medication burden under dose titration according to serial serum calcium measurements and should not be interpreted as elemental calcium intake

During hospitalization, intravenous calcium chloride (5.44 mEq/ampule) was administered as clinically indicated, and acute intravenous calcium use did not differ significantly between groups. After discharge, calcium carbonate was prescribed as 500-mg tablets, with the dose titrated according to serial serum calcium measurements. No significant between-group difference was detected in the prescribed oral calcium tablet burden at discharge or at 3 or 6 months (Fig. 5). All patients also received calcitriol (0.25 micrograms per dose), and no significant between-group difference was observed in calcitriol dose during follow-up.

## Discussion

This retrospective cohort study compared two nonrandomly selected surgical strategies for refractory SHPT. Both TPTX and TPTX + AT achieved satisfactory short-term biochemical control without major perioperative complications, consistent with previous reports [16, 17]. The principal practical finding was that no significant between-group difference in postoperative oral calcium or calcitriol medication burden was detected through 6 months, despite differences in postoperative iPTH and other biochemical markers.

TPTX + AT was associated with higher postoperative iPTH levels, which are compatible with greater postoperative parathyroid activity. However, the present study cannot attribute this activity exclusively to the autograft. Although four glands were identified and confirmed histologically in every patient, this does not exclude an unrecognized supernumerary, ectopic, or microscopic parathyroid focus. Furthermore, routine thymectomy was performed only in the TPTX + AT group. Consequently, the observed iPTH difference may reflect graft function, procedural heterogeneity, or both. Serum phosphate and BALP are indirect measures influenced by dialysis, medication, nutrition, and bone turnover and should not be regarded as graft-specific functional tests [18–20].

The higher treatment-adjusted serum calcium observed in the TPTX group despite lower iPTH was physiologically counterintuitive if serum calcium were interpreted as an untreated endogenous outcome. In this cohort, however, calcium carbonate and calcitriol were actively titrated according to serial measurements. The serum calcium difference therefore may reflect treatment intensity, dialysis-related factors, adherence, or other unmeasured influences and should not be interpreted as superior calcium homeostasis after TPTX. Similarly, the lower BALP in the TPTX group may indicate greater suppression of bone turnover, but BALP alone cannot diagnose adynamic bone disease; bone histomorphometry and additional bone outcomes were not available [1, 21].

A clinically relevant contribution of this study is the quantitative comparison of prescribed oral calcium tablet burden at serial outpatient time points. Previous studies have more commonly reported hypocalcemia, total inpatient calcium use, or the proportion of patients receiving supplementation rather than the actual outpatient oral dose over time [15, 22]. In the present cohort, despite higher postoperative iPTH after TPTX + AT, no significant between-group difference in prescribed oral calcium or calcitriol burden was detected through 6 months. This result should not be interpreted as proof of equivalence because supplementation was individually titrated and the study was not randomized; rather, it provides a descriptive estimate of short-term medication burden under routine clinical care.

SPTX remains an important function-preserving alternative. Although leaving a cervical remnant may create a potential site for persistent or recurrent disease, contemporary evidence indicates that carefully calibrated SPTX can achieve acceptable long-term outcomes [9, 10]. Conversely, TPTX + AT creates an accessible graft site but may permit graft-related hyperplasia, whereas TPTX may increase the likelihood of profound PTH suppression. These trade-offs support a balanced, individualized choice rather than a universal hierarchy among SPTX, TPTX, and TPTX + AT [8–14]. In this context, TPTX alone is increasingly reserved for patients with a lower anticipated need for future transplant-related parathyroid function, such as those with substantial comorbidity or limited transplant candidacy, a pattern reflected in the present cohort, in which TPTX-group patients generally were not pursuing kidney transplantation. TPTX + AT may be considered when preserving accessible, potentially reoperable parathyroid tissue is a priority, for example in patients who may pursue future transplantation.

Long-term recurrence is clinically important but was outside the scope of the present 6-month study. The absence of observed recurrence during this interval does not provide evidence regarding long-term recurrence, and higher short-term iPTH in the TPTX + AT group cannot be used to predict future recurrence. Previous studies have identified graft hyperplasia, cervical remnants, and unrecognized supernumerary or ectopic tissue as potential sources of persistent or recurrent hyperparathyroidism, but these mechanisms require substantially longer follow-up and, when necessary, graft-specific or localization studies [20, 23–26].

Procedure selection should consider transplant plans, comorbidity, life expectancy, the consequences of prolonged hypoparathyroidism, the feasibility of future reintervention, and patient preference. Available guidance does not establish a single optimal operation for all patients, and local surgical expertise and follow-up capability remain relevant [27–29]. Our data support the feasibility of both strategies in selected patients but do not establish superiority of either approach.

## Study limitations

This study has several limitations. First, its retrospective, single-center design, modest sample size, and 6-month follow-up limit generalizability and preclude assessment of long-term recurrence, reoperation, fracture, bone density, quality of life, cardiovascular outcomes, or mortality. Second, procedure allocation was nonrandomized and subject to confounding by indication. Patients undergoing TPTX generally were not pursuing kidney transplantation and may have differed from TPTX + AT patients in comorbidity, candidacy, socioeconomic circumstances, or preference even when measured baseline variables appeared similar. Third, thymectomy was routine in the TPTX + AT group but not in the TPTX group, creating procedural heterogeneity that cannot be separated from the effect of autotransplantation. Fourth, supplementation was titrated rather than fixed; therefore, serum calcium was treatment-mediated, and prescribed tablet count reflects medication burden rather than an untreated physiological calcium requirement. The retrospective medication records did not permit a uniform conversion of the labeled 500-mg calcium carbonate tablet formulation to elemental calcium. Dialysate calcium concentration, dietary intake, adherence, and some medication-related factors were not uniformly available. Fifth, no graft-specific PTH sampling, intraoperative PTH-based completeness assessment, bone histomorphometry, or other test was available to determine the source of postoperative PTH or diagnose adynamic bone disease. Finally, multiple exploratory time-point comparisons were performed without multiplicity adjustment. The results should therefore be interpreted as descriptive and hypothesis-generating.

## Conclusion

TPTX and TPTX + AT both produced satisfactory short-term biochemical control without major perioperative complications in this cohort. TPTX + AT was associated with higher postoperative iPTH, compatible with greater postoperative parathyroid activity; however, no significant between-group difference in prescribed oral calcium or calcitriol supplementation burden was detected through 6 months. Because of nonrandomized allocation, confounding by indication, different thymectomy practices, and limited follow-up, these findings do not establish procedural superiority or long-term recurrence risk.

## Supplementary Information

Below is the link to the electronic supplementary material.

Supplementary Material 1: Table S1. Indications for parathyroidectomy in patients undergoing total parathyroidectomy alone (No-AT) and total parathyroidectomy with autotransplantation (AT). Data are presented as n (%). No p value was calculated for “refractory biochemical SHPT (iPTH >800 with hypercalcemia/hyperphosphatemia despite medical therapy)” because it was an inclusion criterion present in 100% of patients in both groups.No-AT = total parathyroidectomy alone; AT = total parathyroidectomy with autotransplantation; SHPT = secondary hyperparathyroidism; iPTH = intact parathyroid hormone.

## Data Availability

The datasets used and analyzed during the current study are available from the corresponding author upon reasonable request. The data are not publicly available due to restrictions related to patient privacy and institutional ethical approvals.
