# Supplementary material for: Total parathyroidectomy versus total parathyroidectomy with autotransplantation for refractory secondary hyperparathyroidism: short-term biochemical outcomes and postoperative calcium supplementation burden
Source: BMC Endocr Disord. 2026 Jul 21;26:324. doi: 10.1186/s12902-026-02430-8 (PMC13679228; doi:10.1186/s12902-026-02430-8)
Supplement: Supplementary file 1 [file 12902_2026_2430_MOESM1_ESM.docx]

| **Indication** | Total (*N* = 80) | Group | | *p* |
| --- | --- | --- | --- | --- |
|  |  | **No-AT**  (*n* = 40) | **AT**  (*n* = 40) |  |
| Refractory biochemical SHPT (iPTH >800 with hypercalcemia/hyperphosphatemia despite medical therapy)*  Intractable pruritus  Calciphylaxis  Progressive bone pain  Osteitis fibrosa cystica  Pathologic fracture | 80 (100%)  73 (91.3%)  16 (20%)  62 (77.5%)  30 (37.5%)  11 (13.8%) | 40 (100%)  37 (92.5%)  9 (22.5%)  30 (75.0%)  14 (35.0%)  5 (12.5%) | 40 (100%)  36 (90.0%)  7 (17.5%)  32 (80.0%)  16 (40.0%)  6 (15.0%) | NA  1.000  0.781  0.790  0.818  1.000 |

**Table S1. Indications for parathyroidectomy by surgical group**

* No p value was calculated for 'refractory biochemical SHPT' because it was an inclusion criterion present in 100% of patients in both groups.
